# Microglial Activation Damages Dopaminergic Neurons through MMP-2/-9-Mediated Increase of Blood-Brain Barrier Permeability in a Parkinson’s Disease Mouse Model

**DOI:** 10.3390/ijms23052793

**Published:** 2022-03-03

**Authors:** Zhengzheng Ruan, Dongdong Zhang, Ruixue Huang, Wei Sun, Liyan Hou, Jie Zhao, Qingshan Wang

**Affiliations:** 1 School of Public Health, Dalian Medical University, Dalian 116044, China; ruanzz1122@163.com (Z.R.); zddljh@126.com (D.Z.); hrx1994@csu.edu.cn (R.H.); shinysunwei@126.com (W.S.); hou19810103@126.com (L.H.); 2 National-Local Joint Engineering Research Center for Drug-Research and Development (R&D) of Neurodegenerative Diseases, Dalian Medical University, Dalian 116044, China

**Keywords:** blood–brain barrier, microglia, neuroinflammation, rotenone, MMPs

## Abstract

Chronic neuroinflammation has been considered to be involved in the progressive dopaminergic neurodegeneration in Parkinson’s disease (PD). However, the mechanisms remain unknown. Accumulating evidence indicated a key role of the blood–brain barrier (BBB) dysfunction in neurological disorders. This study is designed to elucidate whether chronic neuroinflammation damages dopaminergic neurons through BBB dysfunction by using a rotenone-induced mouse PD model. Results showed that rotenone dose-dependently induced nigral dopaminergic neurodegeneration, which was associated with increased Evans blue content and fibrinogen accumulation as well as reduced expressions of zonula occludens-1 (ZO-1), claudin-5 and occludin, three tight junction proteins for maintaining BBB permeability, in mice, indicating BBB disruption. Rotenone also induced nigral microglial activation. Depletion of microglia or inhibition of microglial activation by PLX3397 or minocycline, respectively, greatly attenuated BBB dysfunction in rotenone-lesioned mice. Mechanistic inquiry revealed that microglia-mediated activation of matrix metalloproteinases-2 and 9 (MMP-2/-9) contributed to rotenone-induced BBB disruption and dopaminergic neurodegeneration. Rotenone-induced activation of MMP-2/-9 was significantly attenuated by microglial depletion and inactivation. Furthermore, inhibition of MMP-2/-9 by a wide-range inhibitor, SB-3CT, abrogated elevation of BBB permeability and simultaneously increased tight junctions expression. Finally, we found that microglial depletion and inactivation as well as inhibition of MMP-2/-9 significantly ameliorated rotenone-elicited nigrostriatal dopaminergic neurodegeneration and motor dysfunction in mice. Altogether, our findings suggested that microglial MMP-2/-9 activation-mediated BBB dysfunction contributed to dopaminergic neurodegeneration in rotenone-induced mouse PD model, providing a novel view for the mechanisms of Parkinsonism.

## 1. Introduction

Chronic exposure to environmental toxins is closely associated with the pathogenesis of Parkinson’s disease (PD) that is characterized by neuronal damage in the nigrostriatal system and formation of Lewy bodies in the surviving dopaminergic neurons [1]. Human studies revealed that chronic exposure to rotenone pesticide significantly elevated the risk of PD [2]. Experimental rodents intoxicated with rotenone also recapitulated the typical features of PD, such as degeneration of nigral dopaminergic neurons, aggregation of α-synuclein, the main component of Lewy bodies, and motor deficits [3]. Although the exact mechanism of PD remains unclear, strong evidence revealed a critical role of chronic neuroinflammation mediated by brain innate immune microglia cells in dopaminergic neurodegeneration [4,5]. Postmortem studies in patients with PD also showed amounts of activated microglia around the damaged dopaminergic neurons [6]. However, how microglia-mediated neuroinflammation damages dopaminergic neurons remains to be elucidated currently.

Accumulating evidence suggested that the disruption of blood–brain barrier (BBB), a complex and dynamic interface between the peripheral blood and the brain, is critical for the pathogenesis of a variety of neurological disorders [7]. A key feature of the anatomical structure of the BBB is the tight junctions of the brain microvascular endothelium, which is modulated by intercellular tight junction (TJ) proteins [8]. Degradation of TJ proteins increases BBB permeability, resulting in brain vasogenic edema, penetration of neurotoxic debris, cells, and microbial pathogens from blood to the brain, and subsequent initiation and activation of multiple pathways of neuronal injury and damage [9,10]. BBB disruption is an important pathological feature in ischemic stroke that contributes to brain vasogenic edema and subsequent neuronal damage [11]. Bill RM and colleagues demonstrated that reducing dynamic relocalization of water channel protein aquaporin 4 expressed in astrocyte, a cell component of neurovascular unit, to BBB reduces brain edema and accelerates functional recovery in rodent models of stroke [12,13]. In patients with PD, Gray and Woulfe found significant increase of serum protein, iron, and erythrocyte extravasation in the putamen, indicating increased BBB permeability [14]. Elevated BBB permeability was also detected in the brain in patients with Alzheimer disease (AD) and other neurological disorders [9]. Moreover, increased BBB integrity by amount of substances, such as MicroRNA-149-5p, coenzymeQ10 [15], DL-3n-butylphthalide [16], and protocatechuic acid [17], was found to be neuroprotective in the pathological neurodegenerative process. These findings revealed that the disruption of BBB integrity contributes to neurodegeneration. However, whether rotenone could induce degeneration of dopaminergic neurons by damaging BBB integrity and related mechanisms remain to be investigated.

For this purpose, C57BL/6 mice received different doses of rotenone for three consecutive weeks and the permeability of BBB and expressions of TJ proteins as well as dopaminergic neurodegeneration were detected. We then investigated the potential mechanisms of how rotenone damages BBB and dopaminergic neurons. Our results suggested that microglial activation-mediated BBB disruption via MMP-2/-9 contributed to dopaminergic neurodegeneration in rotenone-treated mice, providing a novel mechanistic insight for rotenone-elicited neurotoxicity and related Parkinsonism.

## 2. Materials and Methods

### 2.1. Reagents

Rotenone (#R8875) and Evans blue (#E2129) were provided by Sigma-Aldrich, Inc. (St. Louis, MO, USA). The AG RNAex Pro Reagent (#AG21102), Pro Taq HS qPCR Kit, and SYBR Green Premix (#AG11720) were provided by Accurate Biotechnology (Hunan, China). The PLX3397 (#S7818), minocycline (#S4226), and SB-3CT (#S7430) were provided by Selleck (Shanghai, China). Antibodies against tyrosine hydroxylase (TH, #AB152) and ionized calcium binding adaptor molecule-1 (Iba-1, #019-19741) antibodies were provided by EMD Millipore (Temecula, CA, USA) and Wako Chemicals (Richmond, VA, USA), respectively. Antibodies against fibrinogen (#ab34269), zonula occludens-1 (ZO-1, #ab96587), claudin-5 (#ab131259), occludin (#ab167161), MMP-2 (#ab92536), MMP-9 (#ab38898), GAPDH (#ab181602), and the MMP assay kit (#ab112146) were purchased from Abcam (Cambridge, MA, USA). The ECL reagents (#20-500-120) were provided by Biological Industries (Cromwell, CT, USA).

### 2.2. Mice Dosing

Adult male C57BL/6 mice were randomly separated into control, 0.75 mg/kg/d and 1.5 mg/kg/d rotenone groups (*n* = 12). Rotenone was administered to mice by intraperitoneal injection for three consecutive weeks [18,19]. Control mice received equal amounts of vehicle. A second batch of mice were randomly divided into control, rotenone, rotenone + PLX3397, and rotenone + minocycline groups (*n* = 11–12). Rotenone at a dose of 1.5 mg/kg/d was administered as described above. Mice in the rotenone + PLX3397 group received (by gavage, daily) PLX3397 (40 mg/kg/d), a selective antagonist for CSF1R, for 7 days and then were treated every other day 30 min prior to rotenone until the end of the experiment [18,19]. Mice in the rotenone + minocycline group received (i.p.) minocycline (50 mg/kg/d) before 2 days of rotenone injection for 3 weeks (once per day). The doses of PLX3397 and minocycline were selected according to previous reports [20,21]. A third batch of mice were randomly divided into control, rotenone, and rotenone + SB-3CT groups (*n* = 10). Rotenone at 1.5 mg/kg/d was administered as described above. SB-3CT was administered (i.p.) 1 h after rotenone for consecutive 3 weeks (once per day). The dosage of SB-3CT (25 mg/kg/d) was used as reported in previous study [22,23]. All animal procedures and their care were conducted in accordance with the NIH Guide for the Care and Use of Laboratory Animals and were approved by the Institutional Animal Care and Use Committee of Dalian Medical University.

### 2.3. Gait Measurement

The gait performance of mice was detected and analyzed as previously described [24]. The limbs of mice were marked with colored paints and then mice were permitted to walk along a 50 cm-long and 10 cm-wide runway into a dark box. Stride length (the distance between subsequent left-side and right-side forelimb and hindlimbs) and distance (the distance width between forelimbs or between hindlimbs) were measured and the mean value was shown.

### 2.4. Evans Blue Content

Evans blue was administered (i.p.) to mice at a dosage of 80 mg/kg (5 mL/kg). After 4 h of Evans blue injection, mice were perfused by autoclaved PBS transcardially. The brains were quickly removed and the midbrain area was dissected on ice. Brain tissues were homogenized in ice-cold PBS and were then centrifuged at 10,000× *g* for 15 min at 4 °C. The absorbance at 620 nm was detected [25].

### 2.5. Immunohistochemistry

For immunohistochemistry, mice were transcardially perfused with 4% paraformaldehyde (PFA). The dissected brains were then post-fixed for 48 h, followed by 30% sucrose for 2 additional days. Furthermore, the brains were sectioned into free-floating sections of 30 μm thickness. The endogenous peroxidase in the sections was inactivated by 1% H_2_O_2_ at RT. After washing three times with PBS, sections were blocked by 0.4% triton/PBS containing 1% BSA and 4% goat serum, and then were probed with antibodies against TH (1:1000), Iba-1 (1:1000), ZO-1, occludin, or claudin-5 (1:500) at 4 °C for 24 h. The sections were then washed and incubated with biotin-labeled or Alexa-488/594 conjugated (for ZO-1, occludin, or claudin-5) secondary antibodies. For TH or Iba-1 staining, ABC reagents and DAB were added to each section to visualize antibody binding signals. The staining images were acquired (10×/0.25 or 20×/0.5 magnification) by using an Olympus microscope (BX51) with attached digital camera. For ZO-1, occludin, or claudin-5 immunofluorescence staining, the images were acquired at 40× magnification (NA value 0.75).

TH-positive (THir) neurons in the SNpc were quantified by using a microscope [26,27]. In brief, the boundary of SN was outlined based on the mouse brain atlas. A total of 24 consecutive coronal sections for each mouse brain that covers the entire SN area were collected. The first (rostral) and every forth section of the 24 sections of each brain (8 sections for each mouse) were used. The cell count was done by two individuals who were blind to the treatment. The total number of THir neurons in these 8 sections for each mouse was calculated and the results were expressed as the percentage of control mice.

The densities of the TH and Iba-1 staining in the striatum and SN, respectively, were quantified by using ImageJ [27,28]. Briefly, the images prepared from 2–3 brain sections with 120 μm intervals for each mouse were converted into the grayscale picture, and the relative density of the staining was compared based on the total pixels of striatum or SN region (total pixels/area). The quantification of the staining was corrected for background staining by subtracting the pixels without primary antibody.

### 2.6. Real-Time PCR

The midbrain areas were quickly dissected from mice after perfusion with PBS and the total RNA was purified by using Trizol reagents. RNA was reversely transcribed with MuLV reverse transcriptase and oligo dT primers [18,19,29]. SYBR Green Premix and Takara Thermal Cycler Dice Real Time System were used for real-time PCR amplification. Amplification was done at 95 °C for 10 s, 55 °C for 30 s, and 72 °C for 30 s for up to 40 cycles. GAPDH was used to normalize the level of gene mRNA using the 2^−ΔΔCt^ method. The relative alterations (% of control) of mRNA of genes were calculated.

### 2.7. Western Blot

The midbrain areas were quickly dissected from mice after perfusion with PBS and were homogenized [30,31]. After centrifugation at 10,000× *g* for 10 min at 4 °C, the concentrations of protein in the supernatant of homogenates were detected by BCA protein assay kit. The same amounts of protein in each group were loaded to 4–12% SDS-PAGE, and then were transferred to PVDF membranes. A 5% non-fat milk was used to block nonspecific binding before the membranes were probed with primary antibody against fibrinogen, ZO-1, occludin, claudin-5 (1:1000), MMP-2 (1:500), MMP-9 (1:800), or GAPDH (1:4000) at 4 °C for 24 h. Then, the membranes were probed with HRP-linked secondary antibody for 2 h at RT after washing with PBST. The signals of blots were detected by using ECL reagents. The quantification of density of blots was performed based on previous protocol.

### 2.8. Statistical Analysis

All results were shown as mean ± SEM. Results among groups were compared using one-way ANOVA, followed by Tukey’s post hoc testing for pairwise comparisons. The statistic was considered significant at *p* < 0.05.

## 3. Results

### 3.1. Rotenone Increases BBB Permeability

**Figure 1:**
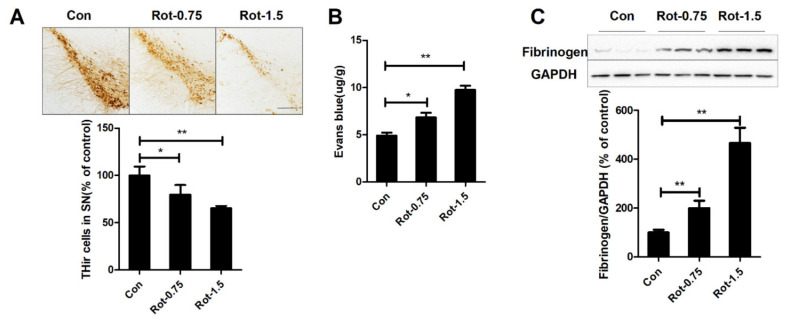
Rotenone dose-dependently damages dopaminergic neurons and elevates BBB permeability in mice. (**A**) Representative images of dopaminergic neuron staining and the number of TH+ dopaminergic neurons in the SNpc of mice for each group. (**B**) The contents of Evans blue in the midbrain of mice were determined. *n* = 3. (**C**) Representative blots of fibrinogen in the midbrain of mice and the quantification of fibrinogen blots. *n* = 6. * *p* < 0.05, ** *p* < 0.01; scale bar = 100 μm. Con: vehicle-treated mice group; Rot-0.75: 0.75 mg/kg/d rotenone-treated mice group; Rot-1.5: 1.5 mg/kg/d rotenone-treated mice group.

To explore whether rotenone could damage dopaminergic neurons through BBB disruption, we initially compared the number of dopaminergic neurons in mice treated with saline, 0.75 mg/kg/d, and 1.5 mg/kg/d rotenone. In agreement with a previous report, rotenone treatment led to significant loss of dopaminergic neurons in mice in a dose-dependent manner (Figure 1A). Subsequently, the brain extravasation of Evans blue was determined to evaluate the BBB permeability. As illustrated in Figure 1B, compared with vehicle controls, rotenone dose-dependently increased the contents of Evans blue in the midbrain of mice. Statistical analysis revealed 38.9% and 98.4% increase of Evans blue in mice injected with 0.75 and 1.5 mg/kg/d rotenone, respectively. Fibrinogen accumulation in the brain is another marker for disrupted BBB. Consistently, elevated expression level of fibrinogen in the midbrain of mice injected with rotenone compared with vehicle controls was detected (Figure 1C).

### 3.2. Rotenone Dose-Dependently Decreases Expression of TJ Proteins in the Substantia Nigra of Mice

**Figure 2:**
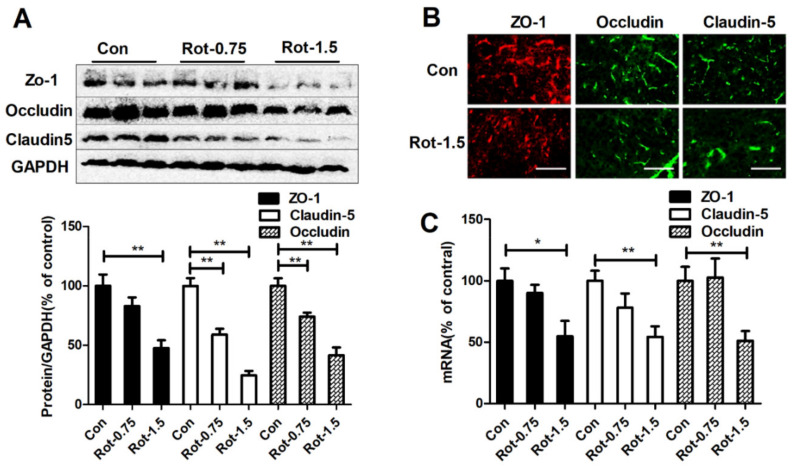
Rotenone dose-dependently reduces expressions and mRNA levels of TJ proteins in mice. (**A**) Representative blots of ZO-1, occludin, and claudin-5 in the midbrain and the quantification of blots. (**B**) Representative images of ZO-1, occludin, and claudin-5 staining in the SN of mice. *n* = 3. (**C**) Gene expressions of ZO-1, claudin-5, and occludin in the midbrain of mice. *n* = 6. * *p* < 0.05, ** *p* < 0.01; scale bar = 100 μm. Con: vehicle-treated mice group; Rot-0.75: 0.75 mg/kg/d rotenone-treated mice group; Rot-1.5: 1.5 mg/kg/d rotenone-treated mice group.

TJ proteins are critical for maintaining the integrity of the BBB. To further confirm whether rotenone could damage the BBB, we compared the expressions of ZO-1, claudin-5, and occludin, three TJ proteins, between mice treated with rotenone and vehicle. Consistent with elevated BBB permeability, marked decrease of ZO-1, claudin-5, and occludin expressions was observed in the midbrain of mice injected with 0.75 and 1.5 mg/kg/d rotenone compared with vehicle (Figure 2A). Immunofluorescence staining further confirmed that 1.5 mg/kg/d rotenone had a significant effect in reducing ZO-1, claudin-5, and occludin expression in the substantia nigra (SN) of mice (Figure 2B). Furthermore, RT-PCR analysis revealed reduced gene levels of ZO-1, claudin-5, and occludin in 1.5 mg/kg/d rotenone-treated mice (Figure 2C). A decreased trend of mRNA levels of TJ proteins was also observed in the 0.75 mg/kg/d rotenone group mice; however, no significant difference was observed (Figure 2C).

### 3.3. Microglial Activation Mediates BBB Disruption in Rotenone-Lesioned Mice

**Figure 3:**
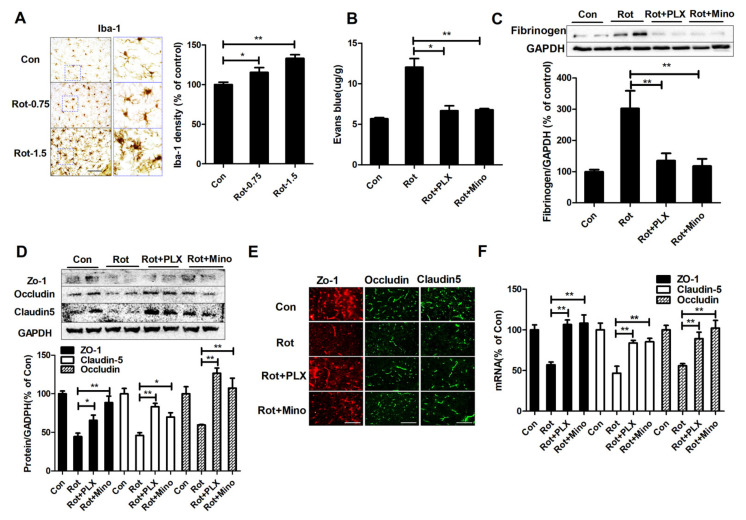
Microglia mediates rotenone-induced BBB dysfunction in mice. (**A**) Representative images of microglial staining and the quantification of Iba-1 staining density. (**B**) The content of Evans blue in mice for each group. (**C**) Representative blots of fibrinogen in mice for each group and the quantification of blots. (**D**) Representative blots of ZO-1, occludin, and claudin-5 in mice for each group and the quantification of blots. (**E**) Representative images of ZO-1, occludin, and claudin-5 staining in the SN of mice. (**F**) Gene expressions of ZO-1, claudin-5, and occludin in the midbrain of mice. *n* = 3–6. * *p* < 0.05, ** *p* < 0.01; scale bar = 100 μm. Con: vehicle-treated mice group; Rot: 1.5 mg/kg/d rotenone-treated mice group; Rot + PLX: 1.5 mg/kg/d rotenone + PLX3397-treated mice group; Rot + Mino: 1.5 mg/kg/d rotenone + minocycline-treated mice group.

Previous study showed that microglia-mediated neuroinflammation contributes to BBB disruption in neurodegenerative diseases [32]. To elucidate the role of microglia in rotenone-induced BBB dysfunction, the effects of rotenone on activation of microglia were firstly examined. As seen in Figure 3A, Iba-1 immunostaining showed that microglia in the control group displayed ramified morphology. While, microglia in rotenone-treated mice displayed hypertrophy morphology and enlarged cell body size, indicating chronically activated microglia. Quantitative analyses also showed increased density of Iba-1 staining in mice lesioned by rotenone compared with vehicle (Figure 3A).

Subsequently, microglial activation was pharmacologically manipulated in the brains of mice, i.e., depletion by PLX3397 and inactivation by minocycline, respectively. In agreement with a previous report [33], PLX3397 and minocycline efficiently depleted Iba-1^+^ microglia and inhibited nigral microglial activation in mice, respectively (Supplementary Figure S1A). The brain penetration of Evans blue was subsequently determined. As seen in Figure 3B, rotenone-induced increase of Evans blue content in mice was significantly dampened by PLX3397 and minocycline. Western blot also showed that microglial depletion and inactivation decreased fibrinogen expression in rotenone-treated mice (Figure 3C). Consistently, Western blot and immunofluorescence staining assay showed that rotenone-induced decrease of ZO-1, claudin-5, and occludin expressions in the SN was abrogated by PLX3397 and minocycline (Figure 3D,E). RT-PCR assay revealed that combined PLX3397/minocycline and rotenone co-treatment also increased mRNA levels of ZO-1, claudin-5, and occludin compared with rotenone alone (Figure 3F).

### 3.4. Microglial Depletion and Inactivation Attenuate MMP-2/-9 Activation in Rotenone-Treated Mice

**Figure 4:**
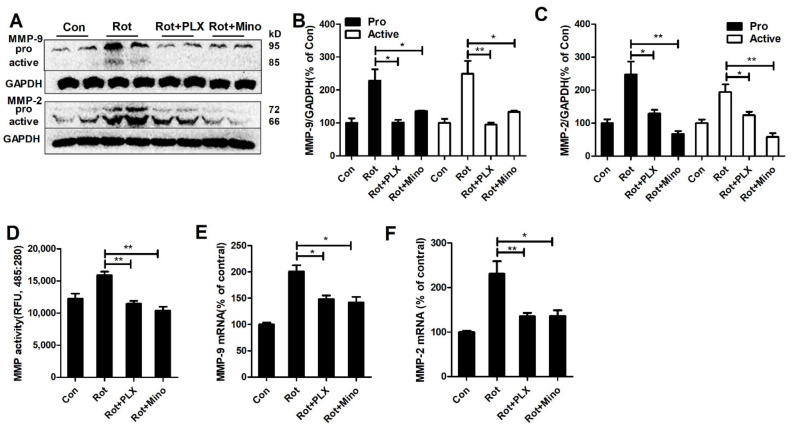
Microglia regulate MMP-2/-9 activation in rotenone-treated mice. (**A**) Representative blots of MMP-2/-9 in mice for each group. (**B**,**C**) The quantification of density of pro- and active MMP-2/-9 blots. (**D**) The activities of MMP in the midbrain of mice for each group. (**E**,**F**) Gene expressions of MMP-2/-9 in the midbrain of mice for each group. *n* = 4–6. * *p* < 0.05, ** *p* < 0.01. Con: vehicle-treated mice group; Rot: 1.5 mg/kg/d rotenone-treated mice group; Rot + PLX: 1.5 mg/kg/d rotenone + PLX3397-treated mice group; Rot + Mino: 1.5 mg/kg/d rotenone + minocycline-treated mice group.

Microglia-mediated neuroinflammation could promote the amount of toxic factors released, in which MMP-2/-9, two members of metalloproteinases family, are critical in degrading extracellular matrix and TJ proteins, leading to BBB disruption. To study whether MMP-2/-9 are involved in microglia-mediated BBB disruption in rotenone-treated mice, the activation of MMP-2/-9 was initially detected with or without microglial depletion and inactivation. In agreement with BBB disruption and reduction of TJ proteins, high levels of MMP-2/-9 expression and activation were observed in rotenone-lesioned mice compared with controls (Figure 4A–C). Interestingly, PLX3397 and minocycline-induced microglial depletion and inactivation, respectively, greatly reduced rotenone-induced expressions of both pro- and activated MMP-2/-9 in mice (Figure 4A–C). The measurement of MMPs activities also revealed a decrease of MMP activation in the midbrain of rotenone-treated mice when co-administrated with PLX3397 and minocycline (Figure 4D). RT-PCR assay showed that rotenone-induced gene transcripts of MMP-2/-9 in mice were also suppressed by PLX3397 and minocycline (Figure 4E,F).

### 3.5. Inhibition of MMP-2/-9 Activation Mitigates Rotenone-Elicited BBB Dysfunction

**Figure 5:**
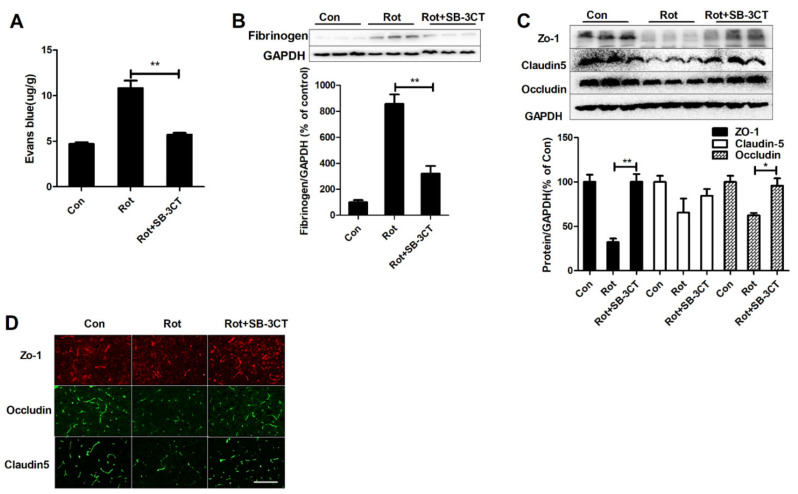
Inhibition of MMP-2/-9 by SB-3CT prevents BBB disruption in rotenone-lesioned mice. (**A**,**B**) The contents of Evans blue in mice for each group. *n* = 3. (**B**) Representative blots of fibrinogen in mice for each group and the quantification of blots. (**C**) Representative blots of ZO-1, occludin, and claudin-5 in mice for each group and the quantification of blots. *n* = 3. (**D**) Representative images of ZO-1, occludin, and claudin-5 staining in the SN of mice. *n* = 3. * *p* < 0.05, ** *p* < 0.01; scale bar = 50 μm. Con: vehicle-treated mice group; Rot: 1.5 mg/kg/d rotenone-treated mice group; Rot + SB-3CT: 1.5 mg/kg/d rotenone + SB-3CT-treated mice group.

To further prove the involvement of MMP-2/-9 in microglial activation-mediated BBB disruption in rotenone-induced mouse model, SB-3CT was administered to mice. As illustrated in Supplementary Figure S2A–C, SB-3CT significantly reduced rotenone-induced MMP-2/-9 activation in mice. Interestingly, SB-3CT-inhibited MMP-2/-9 activation was associated with decreased BBB permeability by showing reduced Evans blue content and fibrinogen expression in rotenone + SB-3CT mice compared with rotenone-alone mice (Figure 5A,B). Western blot showed that SB-3CT also elevated the expressions of ZO-1 and occludin in rotenone-injected mice (Figure 5C). Rotenone-induced reduction of claudin-5 expression was also partially mitigated by SB-3CT, although the difference had no statistical significance (Figure 5C). The recovered TJ proteins in the SN of mice by SB-3CT were further confirmed by immunofluorescence staining (Figure 5D).

### 3.6. Microglial Depletion, Inactivation and Inhibition of MMP2/9 Protect Dopaminergic Neurons in Rotenone-Injected Mice

**Figure 6:**
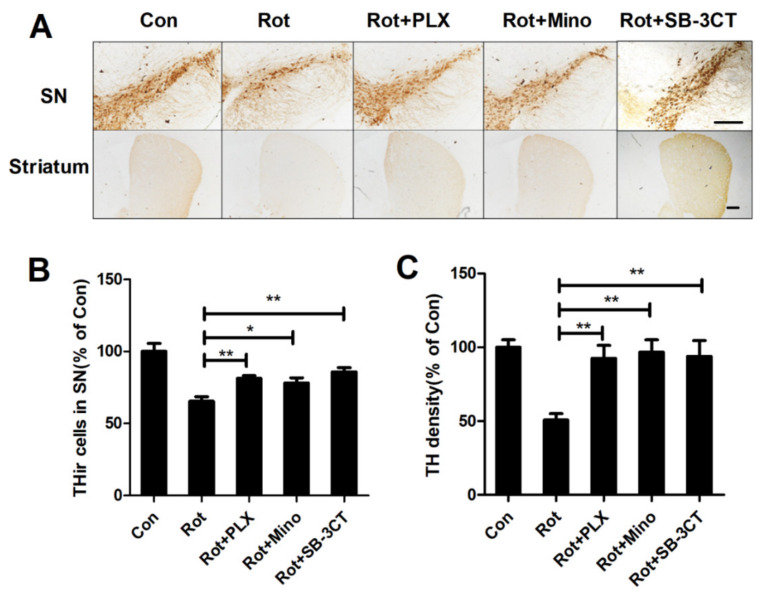
PLX3397, minocycline, and SB-3CT mitigate rotenone-induced dopaminergic neurodegeneration in mice (**A**) Representative images for TH staining in the SN and striatum of mice. (**B**) The quantification of number of TH^+^ neurons in the SNpc. (**C**) The quantification of density of TH staining in the striatum. *n* = 4. * *p* < 0.05, ** *p* < 0.01; scale bar = 200 μm. Con: vehicle-treated mice group; Rot: 1.5 mg/kg/d rotenone-treated mice group; Rot + PLX: 1.5 mg/kg/d rotenone + PLX3397-treated mice group; Rot + Mino: 1.5 mg/kg/d rotenone + minocycline-treated mice group; Rot + SB-3CT: 1.5 mg/kg/d rotenone + SB-3CT-treated mice group.

To determine whether microglia-mediated MMP-2/-9 activation and BBB disruption are associated with dopaminergic neurodegeneration, dopaminergic neurons in the SN and striatum of rotenone-lesioned mice supplemented with or without PLX3397, minocycline, and SB-3CT were quantified by using immunohistochemistry with antibody against TH. Compared with the vehicle, rotenone exposure led to significant loss TH^+^ neurons in the SN of mice, which was markedly blocked by PLX3397, minocycline, and SB-3CT (Figure 6A,B). Consistently, rotenone-induced loss of striatal fibers of dopaminergic neurons was also attenuated by PLX3397, minocycline, and SB-3CT (Figure 6A,C), indicating dopaminergic neuroprotection.

**Figure 7:**
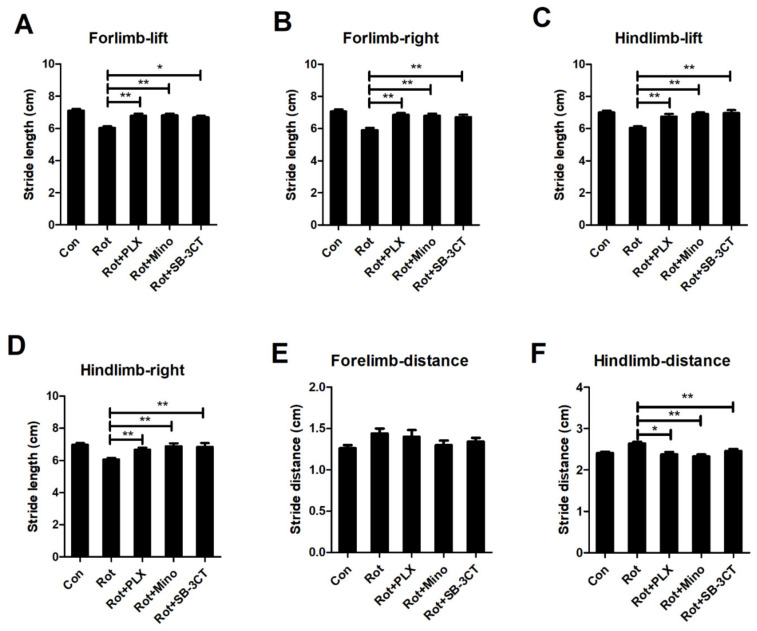
PLX3397, minocycline, and SB-3CT ameliorates rotenone-induced gait abnormality in mice. (**A**–**D**) The stride length between subsequent limb placements (stride length) in mice for each group. (**E**,**F**) The stride distance between limb placements in mice for each group. *n* = 11–12. * *p* < 0.05, ** *p* < 0.01. Con: vehicle-treated mice group; Rot: 1.5 mg/kg/d rotenone-treated mice group; Rot + PLX: 1.5 mg/kg/d rotenone + PLX3397-treated mice group; Rot + Mino: 1.5 mg/kg/d rotenone + minocycline-treated mice group; Rot + SB-3CT: 1.5 mg/kg/d rotenone + SB-3CT-treated mice group.

To determine whether PLX3397, minocycline and SB-3CT-afforded neuroprotection is associated with functional recovery, gait performance of mice was determined. In agreement with dopaminergic neuroprotection, rotenone-induced gait abnormality was significantly ameliorated by PLX3397, minocycline, and SB-3CT as shown by long stride length and short stride distance in PLX3397, minocycline, or SB-3CT and rotenone co-treated mice compared with rotenone-lesioned mice (Figure 7A–F).

## 4. Discussion

In this study, we showed that microglia-mediated BBB disruption via MMP-2/-9 contributed to rotenone-induced dopaminergic neurodegeneration. The following salient features were found: (1) rotenone dose-dependently induced BBB dysfunction; (2) rotenone exposure stimulated microglial activation and microglial depletion or inactivation attenuated rotenone-induced BBB disruption; (3) elevated activation of MMP-2/-9 were observed in rotenone-injected mice, which was significantly dampened through depletion and inactivation of microglia; (4) inhibition of MMP-2/-9 by SB-3CT abrogated rotenone-induced BBB disruption; and (5) microglial depletion, inactivation, or inhibition of MMP-2/-9 attenuated loss of dopaminergic neurons and gait abnormality in rotenone-treated mice.

The disruption of BBB has long been considered to contribute to the pathogenesis of neurodegenerative disorders by releasing vasculature-derived substances to infiltrate the brain, such as fibrinogen [9]. Additionally, BBB breakdown also contributes to environmental toxins-induced neurotoxicity. Disdier et al. found that inhalation exposure to titanium dioxide nano-aerosol induces BBB dysfunction and neuronal synaptophysin decrease in aged rats [34]. Takahashi et al. reported that methylmercury exposure results in severe cerebellar pathological changes in rats by damaging BBB via upregulation of vascular endothelial growth factor expression [35]. In agreement with these findings, we found that rotenone intoxication resulted in increased extravasation of exogenous Evans blue, fibrinogen accumulation, and decreased TJ protein expression in the SN of mice, which was associated with marked loss of dopaminergic neurons.

Our results further revealed that rotenone-induced BBB disruption and dopaminergic neurodegeneration might be related to abnormal microglial activation since microglial depletion or inactivation significantly reduced BBB damage and loss of dopaminergic neurons in rotenone-treated mice. Although microglia are important components of neurovascular units and play key roles in maintaining the structure and function of BBB, over-activated microglia has been reported to damage BBB through the amount of cytotoxic factors released [36]. Lipopolysaccharide (LPS), a bacterial endotoxin identified by the innate immune system, is able to induce BBB breakdown in multiple mouse models of the neurodegenerative process [37,38,39]. Inhibition of neuroinflammatory response significantly elevated BBB integrity and mitigated neurodegeneration. Zhang et al. found that inhibiting toll like receptor-4 (TLR4)-mediated inflammatory pathway by apigenin protects BBB and ameliorates brain injury in a rat model of subarachnoid hemorrhage [40]. Anti-inflammatory compound, curcumin, also prevented BBB damage in focal cerebral ischemic rats, which were associated with decreased mortality, reduced brain edema and infarct volume, as well as ameliorated neurological deficit [41].

MMPs, a family of zinc-dependent endopeptidases, are critical contributors to BBB integrity and brain disease [42]. Among the members, MMP-2/-9 are capable of directly degrading TJ proteins and basement membranes, two components of BBB, leading to increased BBB permeability [43]. It has been demonstrated that microglial activation could damage BBB through release of MMP-2/-9. α-Synuclein dose-dependently elevated expressions and gene transcripts of MMP-9 in primary microglia prepared from rats [44]. Elevated MMP-2/-9 contents and activities were also detected in LPS-stimulated microglia [45]. Inhibition of microglial activation by minocycline reduced BBB injury by blocking activation of MMP-9 in LPS-injected mice [46]. In a severe sepsis rat model, the increase of BBB permeability was associated with elevated expression and activation of MMP-2/-9 in both cortex and hippocampus [47]. Furthermore, pharmacological inhibition of MMP-2/-9 reversed the increase of BBB permeability and improved acute cognitive performance in this rat sepsis model [47]. Here, rotenone exposure increased expression and activation of MMP-2/-9. PLX3397 and minocycline-induced microglial depletion and inactivation, respectively, significantly reduced rotenone-elicited MMP-2/-9 activation. Importantly, inhibition of MMP-2/-9 by SB-3CT markedly increased BBB integrity and attenuated dopaminergic neurodegeneration in rotenone-treated mice. Our findings suggested that MMP-2/-9 released from activated microglia contributed to rotenone-induced BBB dysfunction and neurodegeneration. Consistently, Wu et al. found that lanthanum intoxication impairs the integrity of BBB by reduction of junctional proteins and upregulation of MMP-9 in rats [48]. T-2 toxin, a *Fusarium*-derived cytotoxic fungal secondary metabolite, also induced BBB dysfunction through increased MMP-9 expression and activation [49]. Inhibition of MMP-2/-9 expression by progesterone and its neuroactive metabolite allopregnanolone attenuated BBB disruption and infarct size in a rat model of permanent middle cerebral artery occlusion [50]. It is interestingly to mention that the glymphatic system was also impaired following stroke and other BBB disruptive events. The glymphatic system does not only clear waste products but also solutes, such as lactate and inflammatory cytokines [51]. In addition to subcellular relocalization of aquaporin 4 in astrocyte [52,53], stabilization of BBB through targeting microglial activation could also regulate glymphatic clearance. Feng et al. reported that depletion of microglia via cortical injection of clodronate liposomes resulted in Aβ accumulation through blocking glymphatic clearance in the early stage of an AD mouse model [54]. These findings suggested that targeting microglial activation might provide a new framework for pharmacological intervention in glymphatic clearance in a similar way, such as like targeting astrocytes [13,51].

## 5. Conclusions

In conclusion, this study revealed that rotenone could damage dopaminergic neurons through BBB dysfunction. Furthermore, neuroinflammation mediated by microglia contributed to rotenone-induced BBB impairment and MMP-2/-9 released by activated microglia were identified to be the key factors to mediate microglia-mediated BBB damage and dopaminergic neurodegeneration. These results extended our understanding of the immunopathogenesis of rotenone-induced neurotoxicity and related Parkinsonism. Our study also suggested that increased BBB integrity through inhibition of MMP-2/-9 might be an effective avenue to combat rotenone-induced neurodegeneration.

## Data Availability

All original data are available from the corresponding author upon request.

## Supplementary Materials

The following supporting information can be downloaded at: https://www.mdpi.com/article/10.3390/ijms23052793/s1.

## Abbreviations

AD	Alzheimer disease
BBB	blood–brain barrier
Iba-1	ionized calcium binding adaptor molecule-1
LPS	lipopolysaccharide
MMP-2/-9	metalloproteinases 2 and 9
PD	Parkinson’s disease
PFA	paraformaldehyde
THir	TH-positive
TJ	tight junction
TLR4	toll like receptor-4
ZO-1	zonula occludens-1
