# Supplementary material for: Microglial Activation Damages Dopaminergic Neurons through MMP-2/-9-Mediated Increase of Blood-Brain Barrier Permeability in a Parkinson’s Disease Mouse Model
Source: Int J Mol Sci. 2022 Mar 3;23(5):2793. doi: 10.3390/ijms23052793 (PMC8910886; doi:10.3390/ijms23052793)
Supplement: Supplementary file 1 [file ijms-23-02793-s001.zip › ijms-1590445-supplementary.pdf]

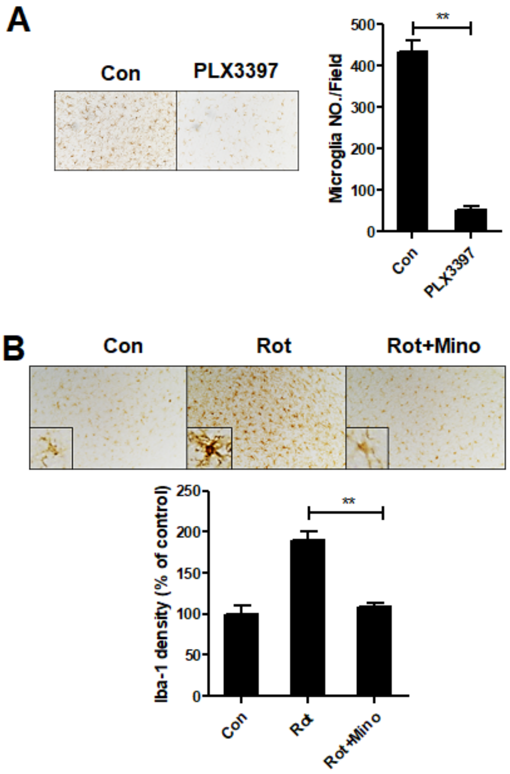

Supplementary Figure S1. The efficiency of PLX3397 and minocycline. (A) Representative images of microglial staining and the number of Iba-1+ microglia in the SN of mice for each group. (B) The representative images of microglial staining and the density of Iba-1 immunostaining in the SN of mice for each group. $^{**}p < 0.01$; Scale bar= 50 µm.

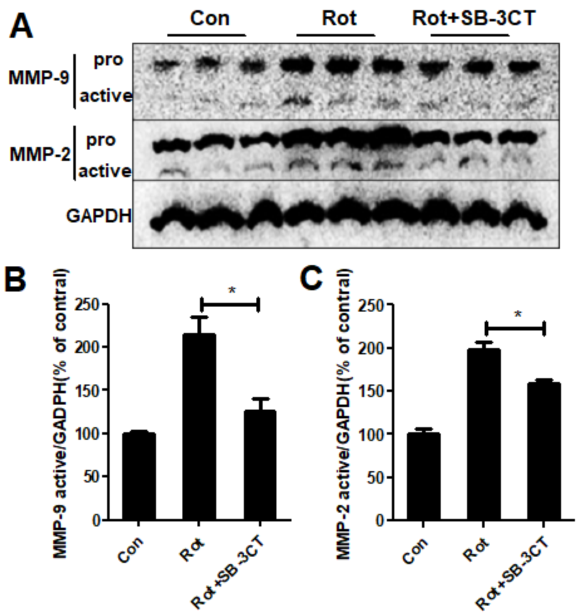

Supplementary Figure S2. SB-3CT blocks rotenone-induced activation of MMP-2/-9 in mice. (A) Representative blots of MMP-2/-9 in mice for each group. (B, C) The quantification of density of active MMP-2/-9 blots. * $p < 0.05$.
